# Waveguide Plasmon Resonance of Arrayed Metallic Nanostructures Patterned on a Soft Substrate by Direct Contact Printing Lithography

**DOI:** 10.3390/s17081867

**Published:** 2017-08-13

**Authors:** Wei-Xiang Su, Chun-Ying Wu, Yung-Chun Lee

**Affiliations:** Department of Mechanical Engineering, National Cheng-Kung University, Tainan 701, Taiwan; koy12345656@gmail.com (W.-X.S.); chunying1009@gmail.com (C.-Y.W.)

**Keywords:** contact printing, arrayed nanostructures, soft polymer substrate, waveguide plasmon resonance

## Abstract

This paper presents a direct contact printing method to obtain arrayed metallic nanostructures on a soft polymer substrate. It utilizes a polydimethylsiloxane (PDMS) mold replicated from silicon molds to transfer metallic nanopatterns onto a polymer substrate based on differences in interfacial bonding energy. Arrayed metallic nanodisks with a disk diameter down to 180 nm and a center-to-center pitch around 400 nm are experimentally patterned on a PET substrate. The patterned metallic nanostructures are then spin-coated with a polymer layer; which mechanically secures the patterned nanostructures and optically allows waveguide plasmon resonance being excited by incident EM waves. Both experimental works and theoretical modeling are given to illustrate the behaviors of different types of plasmon resonance. These arrayed metallic nanostructures patterned on a soft polymer substrate and their tunable optical characteristics open up many possibilities in future engineering applications.

## 1. Introduction

Arrayed metallic nanostructures can interact with incident electromagnetic (EM) waves in several ways and result in intriguing and useful optical spectral transmission, reflection, and absorption characteristics. When subjected to external excitation, free electrons confined in metallic nanostructures can form individually and/or collectively an oscillation with the externally applied electromagnetic field at certain frequency or wavelength. This phenomenon is known as localized surface plasmon resonance (LSPR) and is strongly related to material properties, sizes, shapes, and arrangements of the arrayed metallic nanostructures as well as the material properties of surrounding dielectrics. The LSPR can significantly alter the optical properties in a controllable manner through light-material interaction and hence has found many useful applications in fields such as surface enhanced Raman scattering (SERS) [1,2,3], near-field microscopy [4], surface-enhanced fluorescence [5,6], and molecular sensing [7].

When regularly arrayed metallic nanostructures are deployed next to a dielectric layer or slab, plasmon resonance induced within arrayed nanoparticles can couple to certain eigenmodes of the EM waves travelling inside the dielectric layer/slab waveguide [8]. This new type of waveguide plasmon resonance can further enhance and/or modify the optical spectral characteristics and hence has gained a lot of attention recently [9,10]. To successfully achieve this hybrid waveguide plasmon resonance, the dielectric layer must have a higher optical refraction index than its top and bottom surrounding media and the layer thickness is comparable to the lattice dimension of the arrayed metallic microstructures.

There are many different methods to fabricate arrayed metallic nanostructures. In laboratories, e-beam lithography [11,12,13] is most commonly used approach, but is limited to very low throughput and small patterned area sizes. Advanced photolithography systems used in the semiconductor industry can effectively achieve wafer-size patterning of nanostructures, but the capital investments and running expenses are extremely high. Laser interference lithography [14] provides a viable alternative, however, the choice of lattice patterns is limited and high power laser sources are needed for larger patterning areas. Nano-imprinting lithography is an excellent candidate in terms of patterning area size, achievable feature size, system simplicity, and processing costs. The equipment systems and fabricating processes for nano-imprinting are relatively simple and easier to implement, and hence have been recently used in LSPR research [15]. However, in most of the methods mentioned above for preparing arrayed metallic nanostructures, the substrates used are typically hard solid substrates which in many cases are necessary for making the nanofabrication processes possible. On the other hand, if the substrates are soft, for example polymer substrates, the available nanopatterning methods quickly become very limited due to certain mechanical and chemical limitations of these soft substrates in the fabrication processes.

In this paper, we will demonstrate a direct contact printing method for patterning metallic nanostructures on top of a polymer film. This method is based on our earlier works [16] on contact-transfer patterning with certain modifications aiming at soft polymer substrates. It provides a direct, rapid, easily implemented, and cost-effective method for patterning large-area metallic nanostructures. Section 2 gives details on the fabricating processes as well as experimental results on patterning metallic nanostructures on a polyethylene terephthalate (PET) substrate. After coating a covering layer on these metallic nanostructures, one can achieve different types of waveguide plasma resonance for different applications. In Section 3, numerical modeling of the optical characteristics of fabricated samples is carried out based on finite element method (FEM) to investigate the phenomena of waveguide plasmon resonance. The simulated results are compared with experimental measurements. Conclusions and future perspectives of the proposed contact printing method are given in Section 4.

## 2. Experimental Details and Results 

**Figure 1:**
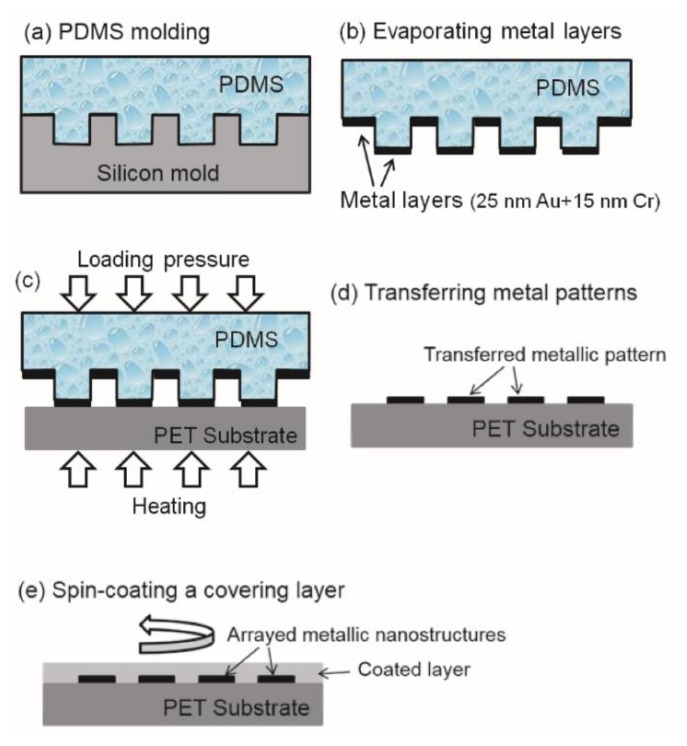
Schematic diagrams of direct contact printing lithography for preparing arrayed metallic nanostructures with a top coating layer: (**a**) PMDS molding from a silicon mold, (**b**) metal layer deposition by e-beam evaporating, (**c**) and (**d**) contact transfer of metallic nano-patterns to a substrate, and (**e**) spin-coating a cover layer as a waveguide for localized surface plasmon resonance.

**Figure 2:**
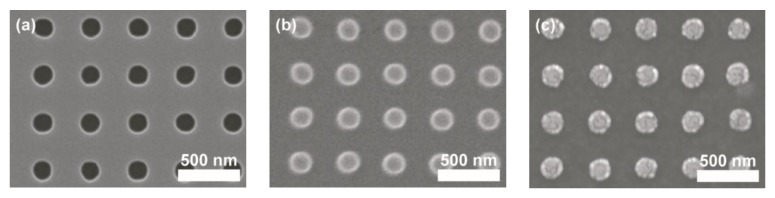
SEM images of (**a**) a silicon mold containing arrayed micro-holes which are 400 nm in center-to-center pitch, 180 nm in hole-diameter, and 230 nm in hole-depth; (**b**) a PDMS mold negatively replicated from the silicon mold; and (**c**) arrayed metallic nano-disks patterned on a PET substrate after contact printing processes.

This section describes the fabrication processes and experimental details for preparing arrayed metallic nanostructures on soft PET substrates for the purpose of generating different types of waveguide plasmon resonance. As shown in Figure 1a, a polydimethylsiloxane (PDMS) mold is first replicated from a silicon mold by standard molding processes [17]. The PDMS material is KER-4690 (Shin-Etsu, Tokyo, Japan) which is cured at an ultraviolet (UV) irradiation of 2000 mJ/cm^2^. The 8” silicon mold is fabricated by a local semiconductor company using standard photolithography methods. On the silicon mold surface, squarely arrayed micro-holes with different dimensions are fabricated to pattern different metallic nano-arrays. There are three different center-to-center pitches of the arrayed holes, namely, 400, 500, and 600 nm. For each pitch, there are three different hole diameters of 180, 250, and 300 nm. Therefore, there are in total nine different samples under investigation. The scanning electron microscope (SEM) image in Figure 2a shows one of the hole-arrays on a silicon mold which has a center-to-center pitch of 400 nm and a hole-diameter of 180 nm, and Figure 2b shows the SEM image of the PDMS mold replicated from this silicon mold.

As shown in Figure 1b, after molding process the PDMS mold surface is deposited with metal layers by an e-beam evaporation system (FSE, Taipei, Taiwan). In this work, a 25 nm thick gold (Au) film and a 10 nm thick chromium (Cr) film are subsequently deposited on the PDMS mold’s surface. Due to the nature of low surface energy of PDMS materials, the deposited metal films are only weakly attached to the PDMS surface. Therefore, when the PDMS mold is brought into contact with a PET substrate and under an externally applied loading pressure and heating temperature, as shown in Figure 1c, the metal films sandwiched in between PDMS mold and PET substrate can be adhered to the PET substrate directly. As shown in Figure 1d, after separating the PDMS mold and the PET substrate, patterned metallic films defined by the convex microstructures of PDMS mold is transferred to the PET substrate. In this work, the applied loading pressure is 80 kPa under a heating temperature of 90 °C for 5 min. As an example, the SEM image of patterned Au/Cr films on a PET substrate using the PDMS mold shown in Figure 2b is given in Figure 2c. Arrayed metallic (Au/Cr) nano-disks with similar characteristic dimensions of the silicon and PDMS molds are successfully obtained based on this contact printing and direct transfer fabrication processes.

Finally, for the purpose of generating waveguide plasmon resonance, the metal-patterned PET substrate is spin-coated with a polymer layer. After solidification of the polymer layer, the patterned Au/Cr nano-disks are firmly sandwiched by the PET substrate and the covering polymer layer. As mentioned above, for waveguide plasmon resonance, the optical refraction index of the coated polymer layer needs to be higher than that of the underlying PET substrate, which is 1.6. In this work, we use a positive tone photoresist (PR) AZ1500 (AZ Electronic Materials, Wiesbaden, Germany) with a thinner PGMEA (TEDIA, Fairfield, CT, USA) at a volume ratio of 1:2 (AZ1500:PGMEA). The refraction index of this PR after curing is 1.7 and is higher than that of PET substrate. At a spinning speed of 3000 rpm, a 250 nm thick PR layer is coated on all the nine samples under testing for waveguide plasmon resonance.

## 3. Numerical Simulation and Optical Measurements

**Figure 3:**
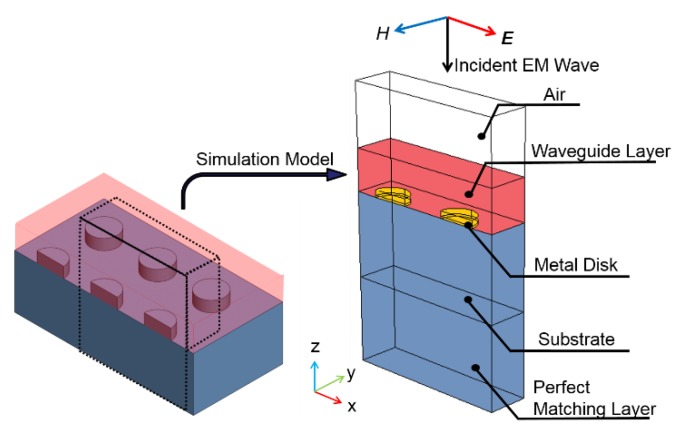
Model used in finite element simulation of optical transmittance of a wave-guided metallic nano-disk array.

The optical transmittance spectrum of these layer-coated and arrayed metallic nanostructures is numerically modeled using a commercial software COMSOL Multiphysics^®^ (COMSOL Inc., Burlington, MA, USA), which has an electromagnetic simulation module based on finite element method. Figure 3 shown the models used in simulating the interaction between an EM wave incident from air, a coated polymer waveguide, squarely arrayed Au/Cr nano-disks, and an underlying PET substrate. The dielectric functions of Au and Cr are described as Drude model [18]. Perfect matching layers are used in the modeling to reduce computing time. Optical transmittance is then calculated as a function of EM wavelength to observe expected plasma resonance phenomena for all the nine samples with 400, 500, and 600 nm center-to-center pitches and 180, 250, and 300 nm nanodisk diameters.

**Figure 4:**
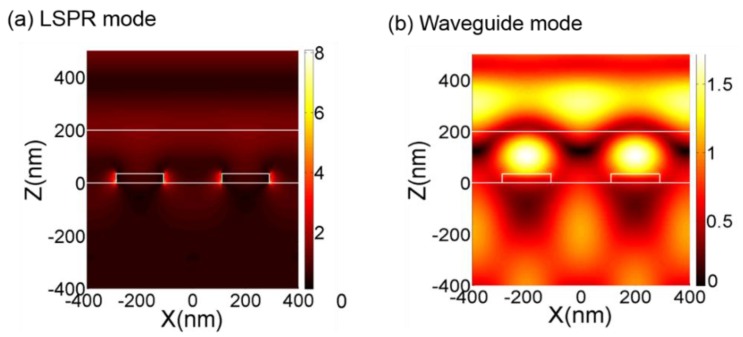
Magnitude of simulated electric field intensity for (**a**) LSPR mode and (**b**) wave-guided mode.

Besides the spectral optical transmittance, the energy distribution of EM waves simulated by COMSOL Multiphysics^®^ can also directly reveal the plasmon resonance phenomenon. For example, Figure 4a,b shows the simulated EM energy intensity distribution within Au/Cr nano-disk arrays without and with a coated PR layer, respectively. The arrayed Au/Cr nanodisks are 400 nm in center-to-center pitch, 180 nm in disk diameter, and 35 nm in disk height (25 nm Au + 10 nm Cr). The thickness of coated PR layer for Figure 4b is 250 nm. In Figure 4a, one can observe the resonance induced between the EM wave and the arrayed metallic nanodisks. In this case, the resonance happens at the wavelength of 710 nm, while in Figure 4b, one can clearly identify a type of waveguide plasmon resonance occurred between adjacent metallic nanodisks and the covering polymer layer, which happens at wavelength of 550 nm in this case.

**Figure 5:**
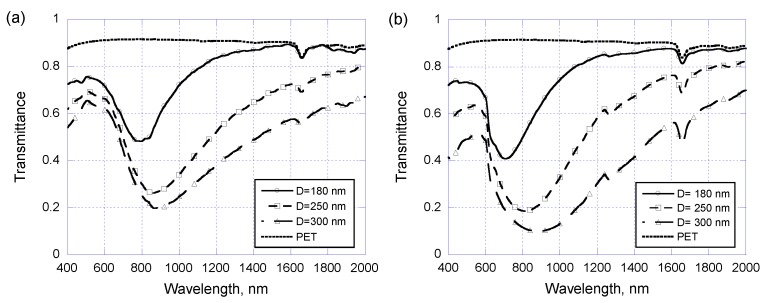
(**a**) Experimental and (**b**) simulated optical transmittance of metallic nano-disk arrays on PET substrates with a center-to-center pitch of 400 nm and three different disk diameters of 180, 250, and 300 nm.

For experimental measurements on these prepared samples, the optical transmittance is measured using a U-3010 spectrophotometer (Hitachi, Tokyo, Japan). An unpolarized light with wavelength *λ_0_* ranging from 400 nm to 2000 nm is normally incident onto the samples and the transmitted power is collected. We start with the arrayed Au/Cr nanodisks patterned on a PET substrate but without a covering PR layer, and the experimentally measured optical transmittance and their theoretically simulated counterparts are displayed in Figure 5a,b, respectively. For demonstration purposes we only show the data for three samples which have a 400 nm center-to-center pitch but different nanodisk diameters of 180, 250, and 300 nm. The optical transmittance of a bare PET substrate is also included in the figures as a reference. From Figure 5a,b the plasma resonance phenomena are indeed observed at wavelength ranging from 800 to 900 nm according to increasing nanodisk diameters from 180 to 300 nm. Increasing nanodisk diameter also reduces the optical transmittance due to less area size for light to pass through. The experimental results agree quite well with the theoretical predictions, and discrepancy may come from variation on nanodisks’ geometry and dimensions. 

**Figure 6:**
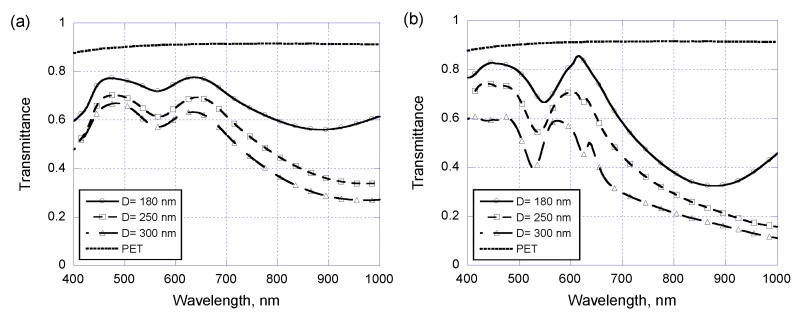
(**a**) Experimental and (**b**) simulated optical transmittance of layer-coated metallic nano-disk arrays on PET substrates with a center-to-center pitch of 400 nm and three different disk diameters of 180, 250, and 300 nm.

Once the arrayed Au/Cr nanodisks are covered with a coated PR layer, waveguide plasmon resonance occurs and dominates the optical performance. As shown in Figure 6a,c are the experimental and theoretical results for the same arrayed Au/Cr nanodisks used in Figure 5 but covered with a 250 nm thick PR layer. It is seen that waveguide plasmon resonance appears at wavelength around 520 to 550 nm, which is mostly determined by the 400 nm center-to-center pitch and the coated PR layer thickness. The nanodisk diameter has little effect on the resonance wavelength, but larger nanodisk diameter will cut down the overall transmittance as intuitively expected.

**Figure 7:**
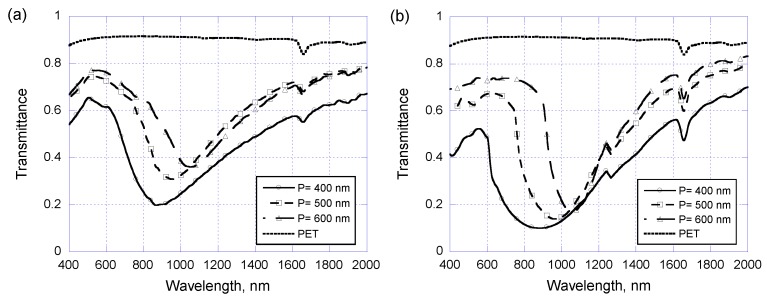
(**a**) Experimental and (**b**) simulated optical transmittance of metallic nano-disk arrays on PET substrates with a disk diameter of 300 nm and three different center-to-center pitch of 400, 500, and 600 nm.

**Figure 8:**
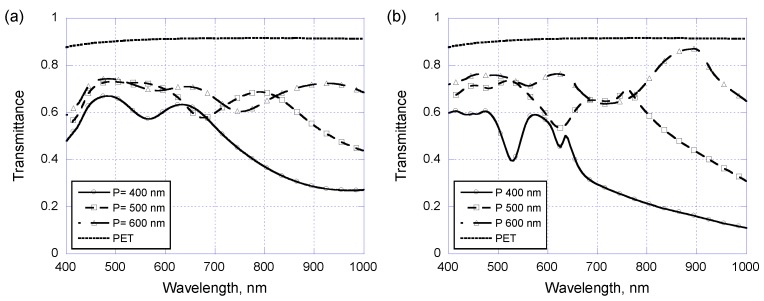
(**a**) Experimental and (**b**) simulated optical transmittance of layer-coated metallic nano-disk arrays on PET substrates with a disk diameter of 300 nm and three different center-to-center pitch of 400, 500, and 600 nm.

In the above discussions concerning the center-to-center pitch of patterned nanodisks applies equally at 400 nm while the nanodisk diameter is changing. Now we are going to see what happens if the nanodisk diameter is kept at 300 nm but the center-to-center pitch is changed from 400 to 600 nm. Again, starting from arrayed Au/Cr nanodisks without covering PR layers and the experimental and theoretical results are shown in Figure 7a and Figure 7b, respectively. Again, the plasmon resonance occurs at wavelength around 900 to 1050 nm when the pitch increasing from 400 nm to 600 nm, as being observed both in experimental measurements and theoretical modeling. Once the arrayed Au/Cr nano-disks are covered with a 250 nm thick PR layer, the experimentally measured optical transmittance and their theoretical counterparts are shown in Figure 8a and Figure 8b, respectively. One can see that the resonance wavelength is now very sensitive to center-to-center pitch as expected for waveguide plasmon resonance. Experimentally, the resonance wavelength is 570, 680, and 750 nm when the pitch is 400, 500, and 600 nm, respectively. Of course, one can also alter the resonance wavelength by simply adjusting the coated PR layer thickness, which is perhaps one of the most important advantages of using waveguide plasmon resonance.

## 4. Conclusions

In this study, we demonstrate a rapid, large-area, easily implemented, and low-cost fabrication method to obtain arrayed metallic nanostructures on soft polymer substrates. Arrayed metallic (Au/Cr) nanodisks with a disk diameter down to 180 nm and a center-to-center pitch down to 400 nm are experimentally demonstrated on a PET substrate using this direct contact printing method. The adhesion force between metal and polymer substrate play a key role in this fabrication method as well as the low surface energy of PDMS materials, the conformal contact pressure, and the heating temperature which brings the polymer substrate close to its glass transition status. The smallest feature size and the largest patterning area size that can be achieved by this method are mostly determined by the silicon mold prepared from standard photolithograph method. In this work, the silicon mold is an eight-inch wafer with the capability of 130 nm in linewidth.

The patterned metallic nanostructures can be spin-coated with a polymer layer such as a photo-resist layer. The covering polymer layer serves two purposes. Mechanically it offers a protection layer to secure patterned nanostructures on PET substrates. Optically it serves a waveguide on top of the arrayed metallic nanostructures so that waveguide plasmon resonance can be excited by incident EM waves. The optical refraction index of the covering layer ought to be larger than that of the substrate, and the layer thickness can be chosen to control the optical characteristics of the waveguide plasmon resonance. Both experimental works and theoretical modeling are given in this paper to illustrate the behaviors of different types of plasmon resonance. It is conceivable that such a soft and polymer-based metallic nano-arrays and their tunable optical characteristics can find many applications in soft and wearable optoelectronics devices in the future.
